# Urea Output by L_3_ *Teladorsagia circumcincta* and some Properties of Two Urea Producing Enzymes

**Published:** 2013

**Authors:** N Muhamad, LR Walker, DC Simcock, KC Pedley, HV Simpson, S Brown

**Affiliations:** 1 Universiti Kuala Lumpur, Royal College of Medicine Perak, 3 Greentown Road, 30450 Ipoh, Perak, Malaysia; 2 Institute of Food, Nutrition and Human Health, Massey University, Private Bag, Palmerston North, New Zealand; 3 Institute of Veterinary and Biomedical Sciences, Massey University, Private Bag 11222, Palmerston North, New Zealand; 4 School of Human Life Sciences, University of Tasmania, Locked Bag 1320, Launceston, Tasmania 7250, Australia

**Keywords:** Kinetics, *Teladorsagia circumcincta*, Urea

## Abstract

**Background:**

Like several other parasites, *Teladorsagia circumcincta* secretes or excretes urea, but neither the rate of efflux nor the possible metabolic sources of the urea has been considered.

**Methods:**

Parasites were maintained by passage through sheep. Urea efflux was measured using phenol/hypochlorite after treatment with urea aminohydrolase. The kinetics of creatine amidinohydrolase and arginine amidinohydrolase were characterised by coupling the reactions with urea aminohydrolase and glutamate dehydrogenase.

**Results:**

Infective L_3_ *T. circumcincta* secreted or excreted urea at 25% of the rate of NH_3_/NH_4_^+^. The rate of urea efflux was about 84 pmol h^−1^ (10^3^ larvae)^−1^ over 4 hours, corresponding to about 11 nmol h^−1^ mg^−1^ protein. We could not detect urea aminohydrolase activity, but urea production by both creatine amidinohydrolase and arginine amidinohydrolase could be detected. The apparent *K*_m_ and *V*_max_ of creatine amidinohydrolase were 1.1 mM and 48 nmol h^−1^ mg^−1^ protein, respectively, and the activity was greatest at pH 8. The apparent *K*_m_ and *V*_max_ of arginine amidinohydrolase were 0.7 mM and 62 nmol h^−1^ mg^−1^ protein, respectively, and the activity was greatest at pH 7.9.

**Conclusion:**

The activity of creatine amidinohydrolase and arginine amidinohydrolase was sufficient to account for the rate of urea secretion or excretion.

## Introduction

A number of nitrogenous small molecules are known to be secreted or excreted by nematodes. Of these, NH_3_/NH_4_^+^ and urea are probably the most commonly observed. Urea secretion or excretion has been reported in *Ascaris lumbricoides*, *Ascaridia galli*, *Nematodirus* spp. and *Panagrellus redivivus*, but was not observed in *Trichinella spiralis*, *Nippostrongylus brasiliensis*, *Ditylenchus triformis*, or, to any significant extent, in *Caenorhabditis briggsae* (1). In the case of *A. lumbricoides* the rate of urea secretion or excretion is slower than that of NH_3_/NH_4_^+^ (2–4). We have shown previously that L_3_ *Teladorsagia circumcincta* secretes or excretes NH_3_/NH_4_^+^ at 0.18-0.6 pmol h^−1^ larva^−1^ (5, 6) and, while urea secretion or excretion has also been reported for L_3_ *T. circumcincta*, no supporting data were shown and no indication of the rate was given (7).

The source of the urea secreted or excreted by *T. circumcincta* has not yet been investigated in any detail. Of the enzyme reactions known to involve urea, Rogers (2) reported activity of urea aminohydrolase in *A. lumbricoides* and *Nematodirus* spp. and a much lower activity in *Haemonchus contortus*. He also reported arginine amidinohydrolase activity in *A. lumbricoides*, *Nematodirus* spp. and *A. galli* (2) and it has been reported in *T. circumcincta* although no data were given (8, 9), but not in *Toxoplasma gondii* (10, 11). Paltridge and Janssens (4) confirmed that arginine amidinohydrolase activity could be observed in *A. lumbricoides*, but of the remaining urea cycle enzymes, only ornithine carbamoyltransferase (E. C. 2.1.3.3) was detectable.

Here we report on the initial kinetics of secretion or excretion of urea by L_3_ *T. circumcincta*. We also attempted to assay urea aminohydrolase (E. C. 3.5.1.5)

1 $$\text{urea}+{\text{H}}_{2}\text{O}⇌{\text{CO}}_{2}+2{\text{NH}}_{3}$$

to determine whether the apparent rate of urea secretion or excretion might have been influenced by the activity of this enzyme. We also quantified some of the properties of two urea producing enzymes: arginine amidinohydrolase (E. C. 3.5.3.1)

2 $$\text{arginine}+{\text{H}}_{2}\text{O}⇌\text{ornithine}+\text{urea}$$

and creatine amidinohydrolase (E. C. 3.5.3.3)

3 $$\text{creatine}+{\text{H}}_{2}\text{O}⇌\text{sarcosine}+\text{urea}.$$

We show that the activity of either of these amidinohydrolases (eqns 2-3) would be sufficient in the absence of any detectable activity of urea aminohydrolase (eqn 1) to account for the observed rate of secretion or excretion of urea.

## Materials and Methods

### Parasite culture and homogenate preparation

Pure strains of *T*. *circumcincta* were maintained by regular passage through sheep to provide L_3_. Larvae were concentrated by centrifugation at approximately 1000 × *g*, washed and then resuspended in 100 mM KH_2_PO_4_-KOH pH 7.5. Where necessary adult *T. circumcincta* were obtained from the abomasal contents of donor sheep as described previously (12). It was not practicable to culture sufficient L_3_ or obtain enough adults to purify the enzymes and so all the work described here was carried out using nematode homogenates. In order to homogenise the nematodes, the suspension was frozen at −20°C and then ground manually at 4°C as described previously (13).

### Ammonia and urea determination

The concentration of NH_3_/NH_4_^+^ in was determined spectrophotometrically at 635 nm after reaction with hypochlorite and phenol (14). Concentrations were determined by reference to an NH_4_Cl standard.

### Ammonia and urea excretion or secretion

The excretion or secretion of NH_3_/NH_4_^+^ and urea was monitored at 37°C in capped Eppendorf tubes. The tubes containing 50000 L_3_ mL^−1^ in 1 mL 0.8 mM NaH_2_PO_4_-NaOH pH 7.0. Before the concentration of NH_3_/NH_4_^+^ was determined, the incubation tubes were centrifuged briefly to pellet the larvae and the supernatant was adjusted to pH 7.

To determine the efflux of urea, 1 U urea aminohydrolase was added to 0.5 mL of the supernatant which was incubated at 37°C for 30 min, after which total NH_3_/NH_4_^+^ was determined. The urea produced was estimated as half of the difference between the total NH_3_/NH_4_^+^ measured after urea hydrolysis and the NH_3_/NH_4_^+^ determined without enzymatic treatment.

### Enzyme activities

By using urea aminohydrolase to hydolyse the urea produced by creatine or arginine amidinohydrolase and then employing the reductive amination reaction of glutamate dehydrogenase to incorporate the NH_4_^+^ into glutamate it was possible to monitor the oxidation of NADH at 340 nm. Using S to denote the substrate (arginine or creatine) and P to indicate the corresponding product (ornithine or sarcosine) of the amidinohydrolase (eqns 2-3), these reactions can be written

S + H_2_O ⇌ P + urea

urea + H_2_O ⇌ CO_2_ + 2NH_3_

2 α-ketoglutarate + 2 NH_3_ + 2 NADH ⇌ 2 glutamate + 2 NAD^+^ + 2 H_2_O,

so the observed rate of NADH oxidation was assumed to be twice the rate of the amidinohydrolase reaction. Details of the assays are provided in the relevant figure legends Two approaches were employed to attempt to measure the activity of endogenous urea aminohydrolase. The first coupled the production of NH_4_^+^ to the reductive amination reaction of glutamate dehydrogenase in which the oxidation of NADH was monitored at 340 nm. The second approach involved detecting the NH_3_/NH_4_^+^ directly using the chemical assay described in section 2.2.

The protein concentration of homogenates was determined using the Bradford method (15) and bovine serum albumin as the standard. No detergents were employed to solubilise the nematode homogenate as they can interfere with protein assays (16).

### Analysis

While it is likely that the kinetics of L_3_ *T. circumcincta* creatine amidinohydrolase and arginine amidinohydrolase can be modelled using the same mechanism as that of other species (17–19), we have not attempted to employ these models in the analysis presented here. Instead, apparent *K*_m_s and *V*_max_s were obtained by fitting the standard Michaelis-Menten expression to the activity data for each substrate. This approach is consistent with that adopted previously (20, 21). Estimates of apparent *V*_max_ depend on the enzyme concentration, so comparisons between L_3_ and adult should be made with caution, but such comparisons can be made more reliably for estimates involving the same lifecycle stage. Estimates of apparent *K*_m_ are independent of enzyme concentration, so comparisons between values for L_3_ and adult preparations can be made with more confidence.

All nonlinear regression and the other analyses described below were carried out using R (22).

## Results

### Accumulation of NH_3_/NH_4_^+^ and urea

**Fig. 1:**
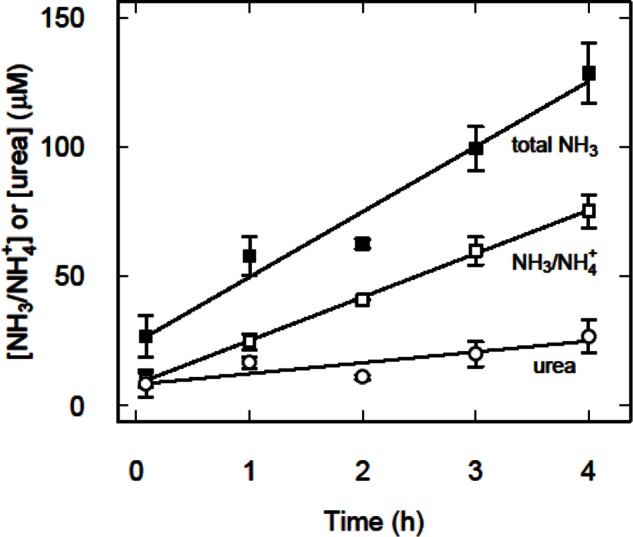
Initial accumulation of total NH_3_ (■), NH_3_/NH_4_^+^ (□) and urea (◯) in suspensions of 50000 larvae mL^−1^ in 0.8 mM KH_2_PO_4_-KOH pH 7 incubated in sealed Eppendorf tubes at 37°C. Total NH_3_ is the concentration of NH_3_/NH_4_^+^ measured in the medium after treatment with urea aminohydrolase. Half of the difference between total NH_3_ and the concentration of NH_3_/NH_4_^+^ observed without enzyme treatment was used to estimate the urea concentration. The solid lines are the least squares fit to all of the appropriate data. The error bars represent ± SEM (*n* = 3)

As we have previously reported (5, 7) NH_3_/NH_4_^+^ accumulated in a medium containing L_3_ *T. circumcincta*. However, treatment of the medium with urea aminohydrolase increased the total concentration of NH_3_/NH_4_^+^ (Fig. 1), from which we infer that urea was also secreted or excreted by the larvae.

Within 5 min of setting up the suspensions of larvae, the concentration of NH_3_/NH_4_^+^ was 8 ± 3 µM (*P* = 0.03), which was increased to 24 ± 7 µM (*P* = 0.005) by treatment with urea aminohydrolase (Fig. 1). From this we estimated that the corresponding concentration of urea was about 8 µM. Thereafter, the rate of NH_3_/NH_4_^+^ accumulation was 0.336 pmol h^−1^ larva^−1^ and this was increased by about 0.17 pmol h^−1^ larva^−1^ by urea aminohydrolase treatment, both of which were approximately constant for the first four hours of incubation. From the difference we infer that there was a constant rate of urea accumulation of 0.084 pmol h^−1^ larva^−1^ over this time (Fig. 1).

### Enzymatic reactions involving urea

Sustained attempts to measure endogenous urea aminohydrolase activity were unsuccessful. Neither the enzymatic assay, in which NH_3_/NH_4_^+^ production was monitored using glutamate dehydrogenase, nor the chemical method, involving the measurement of NH_3_/NH_4_^+^ production using hypochlorite and phenol (14), yielded any evidence of the activity of this enzyme. Of course, this was important in our efforts to monitor urea secretion or excretion and the activity of both arginine and creatine amidinohydrolase.

**Fig. 2:**
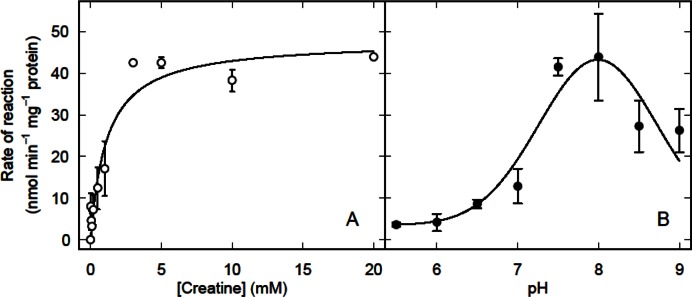
Rate of creatine hydrolysis in homogenates of L_3_ *T. circumcincta* as a function of creatine concentration (A) and pH (B). In (A) the smooth curve is the least squares fits of the Michaelis-Menten equation to all the data. The error bars represent ± SEM for *n* = 4 (A) or *n* = 2-5 (B). The reactions were carried out at 30°C using 50 µM α-ketoglutarate, 0.2 mM NADH, 1 U urea aminohydrolase, 1 U glutamate dehydrogenase and 50 µg of homogenate protein in in 100 mM phosphate buffer. In (B) 5 mM creatine was used

The activity of creatine amidinohydrolase was small (*V*_max_ = 48 ± 2 nmol min^−1^ mg^−1^ protein) and the apparent *K*_m_ was 1.1 ± 0.1 mM (Fig. 2A).

The activity was greatest at pH 8 and declined at more acidic and alkaline pHs with apparent p*K*_*a*_s of 7.1 and 8.9 (Figure 2B). The activity of creatine amidinohydrolase was not affected by Fe(II), but was strongly inhibited by Fe(III), Mg(II), and Cu(II) (Table 1). The activity was slightly stimulated by 2 mM EDTA, but neither ATP nor ADP had any significant effect (Table 1).

**Table 1:** Effect of ions and adenine nucleotides on the activity of creatine amidinohydrolase in homogenates of L_3_ *T. circumcincta*

Conditions	Rate of creatine hydrolysis (nmol min^−1^ mg^−1^ protein) (%)	
Control	42	(100)
+ 0.5 mM FeSO_4_	40	(95)
+ 0.5 mM FeCl_3_	7	(17)
+ 0.5 mM CuSO_4_	5	(12)
+ 0.5 mM MgCl_2_	12	(28)
+ 2 mM EDTA	52	(124)
+ 0.5 mM ATP	40	(95)
+ 0.5 mM ADP	43	(102)

In homogenates of adult nematodes (*n* = 2), creatine amidinohydrolase appeared to have kinetic properties that were very similar to those of the L_3_ enzyme shown in Figure 2A. For the adult enzyme, the apparent *V*_max_ was 45 ± 3 nmol min^−1^ mg^−1^ protein and the apparent *K*_m_ was 0.6 ± 0.1 mM (data not shown).

**Fig. 3:**
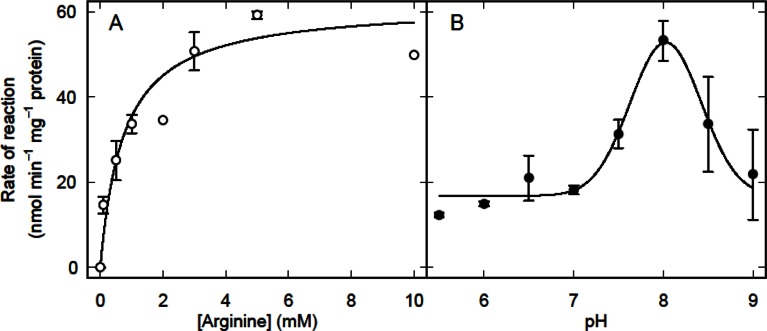
Rate of arginine hydrolysis in homogenates of L_3_ *T. circumcincta* as a function of arginine concentration (A) or pH (B). The smooth curve in (A) is the least squares fit of the Michaelis-Menten equation to all the data. The error bars represent ± SEM for *n* = 3 (A) or *n* = 2-5. The reactions were carried out at 30°C using 100 µM α-ketoglutarate, 0.2 mM NADH, 1 U urea aminohydrolase, 1 U glutamate dehydrogenase and 50 µg of homogenate protein in 100 mM phosphate buffer. In (B) 5 mM arginine was used

In L_3_ *T. circumcincta* the activity of arginine amidinohydrolase was also small (*V*_max_ = 62 ± 4 nmol min^−1^ mg^−1^ protein) and the apparent *K*_m_ was 0.7 ± 0.2 mM (Figure 3A), although for adults the *V*_max_ was 126 ± 6 nmol min^−1^ mg^−1^ protein and the apparent *K*_m_ was 1.4 ± 0.2 mM (*n* = 2). The activity in homogenates of L_3_ nematodes was greatest at pH 7.9 and declined at more acidic and alkaline pHs with apparent p*K*_*a*_s of 7.4 and 8.7 (Figure 3B). The rate of arginine hydrolysis was halved by 0.1 mM Fe(II) and Mn(II) stimulated the activity slightly (Table 2), consistent with there being no significant loss from the enzyme of the Mn(II) required for activity (23). Low concentrations of Cu(II) or EDTA stimulated the activity of the enzyme, but slightly higher concentrations were inhibitory (Table 2).

**Table 2:** Effect of ions on the activity of arginine hydrolysis by L_3_ arginine amidinohydrolase in homogenates of L_3_ *T. circumcincta*

Conditions	Rate of arginine hydrolysis (nmol min^−1^ mg^−1^ protein) (%)	
Control	41	(100)
+ 0.1 mM FeSO_4_	19	(46)
+ 1 mM CuSO_4_	30	(73)
+ 0.1 mM CuSO_4_	48	(117)
+ 0.1 mM MnCl_2_	43	(105)
+ 1 mM MnCl_2_	46	(112)
+ 0.1 mM EDTA	45	(124)
+ 1 mM EDTA	32	(78)

## Discussion

We have shown that the rate of urea secretion or excretion by L_3_ *T. circumcincta* was similar to those reported by Rogers (2) for *Nematodirus* spp., but higher than those he reported for *A. lumbricoides* and *A. galli* (Table 3). The rate of urea secretion or excretion by *T. circumcincta* was about 25% of that of NH_3_/NH_4_^+^, which is similar to previous reports (2–4) for *A. lumbricoides*, *Nematodirus* spp. and *A. galli* (Table 3). We have also shown that L_3_ *T. circumcincta* have both arginine and creatine amidinohydrolase activity, but we were unable to detect any urea aminohydrolase activity.

**Table 3:** Rates of secretion or excretion of urea and NH_3_/NH_4_^+^ reported for various parasites

Species	Rate of secretion or excretion (nmol h^−1^ g^−1^ WW)		Relative rate	Reference
	urea	NH_3_/NH_4_^+^	(urea: NH_3_/NH_4_^+^)	
*A. lumbricoides*	–	200	–	(29)
*A. lumbricoides*	17	80	0.21	(3)
*A. lumbricoides*	52	524	0.10	(4)
*Nematodirus* spp.	312	–	–	(2)
*A. galli*	89	–	–	(2)
*T. circumcincta*	252	1008	0.25	this work

The presence of arginine amidinohydrolase activity is consistent with the observations of activity in *A. lumbricoides*, *Nematodirus* spp. and *A. galli* (2, 4). It is possible that arginine deiminase (E. C. 3.5.3.6), which hydrolyses arginine to citrulline and NH_3_/NH_4_^+^, might also be active in L_3_ *T. circumcincta* homogenates. However, we think this is unlikely for two reasons. First, control reactions in which the urea aminohydrolase was omitted yielded very little activity, perhaps consistent with the low rates of chemical hydrolysis previously reported (24). Second, while there is also an orthologue of the human arginine amidinohydrolase gene in the *Caenorhabditis elegans* genome (T24F4.1 or NP_508948), we were unable to identify an arginine deiminase homologue in the *C. elegans* genome.

Our observation of creatine amidinohydrolase activity may be novel. We have been unable to identify either a previous report of this activity in a nematode or a creatine amidinohydrolase homologue in the *C. elegans* genome or among the sequences currently available for other nematodes. The maximum activity of creatine amidinohydrolase was observed at the same pH as that of the enzyme from *Pseudomonas putida* (25) and *Alcaligenes* sp. (20). However, the apparent *K*_m_ for creatine is much more similar to that of the *P. putida* enzyme (*K*_m_ = 1.33 mM, (26)) than that of the *Alcaligenes* sp. enzyme (*K*_m_ = 17.2 mM, (20)), although Schumann *et al*. (27) gives an estimate of 14.3 mM for the *P. putida* enzyme. The effects of Fe(II), Cu(II) and EDTA were similar to those reported for the enzyme from *Alcaligenes* sp. and *Arthrobacter ureafaciens* (20, 28), although Mg(II) had no effect on the *Alcaligenes* sp. enzyme (20). The crystal structure of the *P. putida* enzyme shows that the creatine is stabilised at the carboxyl end by a pair of arginines and at the guanidino nitrogen end by a pair of glutamate residues, and the substrate is sandwiched between a phenylalanine residue and a catalytically significant histidine (18). So, the lower p*K*_*a*_ in the pH dependence (Fig. 2B) might reflect changes in the protonation of either the glutamates or the histidine and the upper p*K*_*a*_ probably reflects pH-induced changes in the polarity of the guanidinium group.

Our inability to detect urea aminohydrolase activity may simply reflect a failure to identify the right assay conditions or test the parasite at the appropriate stage in its life cycle. Urea aminohydrolase activity has been identified in *A. lumbricoides*, *H. contortus* and *Nematodirus* spp. (2) and nucleotide sequence similar to that encoding the β subunit of the enzyme from *Pseudomonas fluorescens* has been identified in the *Caenorhabditis elegans* genome (hypothetical protein F40G9.5, GenBank accession code: ACO15784.1). However, Rogers (2) reported that the urea aminohydrolase activity was much lower in *H. contortus* than in *A. lumbricoides* and *Nematodirus* spp. and concluded that it was unlikely to protect the nematode from urea in the host. If urea aminohydrolase is present, we conclude that the activity is unlikely to have significant impact on the rate at which urea is secreted or excreted by L_3_ *T. circumcincta*.

The rate of urea secretion or excretion was about 0.084 pmol h^−1^ larva^−1^ which corresponds to about 11 nmol h^−1^ mg^−1^ protein (5). The apparent *V*_max_s for creatine and arginine amidinohydrolase are 48 and 62 nmol h^−1^ mg^−1^ protein, respectively, so either enzyme could account for the observed urea output. Moreover, the enzymes have similar apparent *K*_m_s, so the most significant factor in determining their relative contribution to urea efflux is the intracellular concentration of each substrate.

## Conclusion

The activity of creatine amidinohydrolase and arginine amidinohydrolase was sufficient to account for the rate of urea secretion or excretion.
